# Evocation to the Dr. Carlos J. Finlay Barres on the centennial of his death

**Published:** 2016-03-30

**Authors:** Víctor Guillermo Ferreira Moreno

**Affiliations:** Departamento de Radiología. Hospital Pediátrico de Matanzas. Universidad de Ciencias Médicas de Matanzas. Cuba

**Keywords:** Carlos J. Finlay, yellow fever, discovery, history of the medicine

## Abstract

About the editorial of the Professor Guillermo Llanos: "Carlos J. Finlay: the forgotten Pasteur of America", a hundred years after his death and through a documental review, a summary of the life and work of this great man of science was conducted. Finlay was a notable figure of the American medicine, he conceived a new infection way able to explain the propagation of the yellow fever, and added the possibility of their scientific confirmation by an experimental method. For all the above-mentioned Finlay was recognized as the humanity's benefactor

## Abstract (es)

A propósito del editorial del Profesor Guillermo Llanos: "Carlos J. Finlay: el Pasteur olvidado de América", a cien años de su fallecimiento y a través de una revisión documental, se realizó una síntesis de la vida y obra de ese gran hombre de ciencias, figura insigne de la medicina americana y benefactor de la humanidad, quien concibió una nueva forma de contagio capaz de explicar la propagación de la fiebre amarilla, y le adicionó la posibilidad de su comprobación científica mediante un método experimental.

## Introduction

Human development is the complement of all efforts, all work and all investigations. Each in his time served as a starting point for new discoveries and new advances. To seek to subtract merit to someone in their historical moment, it is imprudent and a lack of critic sense. The truly fair and honest is to accept the historical facts in all they have of greatness and to grant them the recognition and the glory that deserve. Was Finlay (Camagüey 12/03/1833 - 08/20/1915 Havana) 1,2 who had the brilliant idea of the discovery and the conception of the actual way of yellow fever transmission, exercising in the middle of miseries and misunderstandings, but with such personality, that never allowed that the limited resources of their patients deprives them the aid of his science and on the other hand, without abandoning his scientific work in spite of his own hardships. On the occasion of one of the last anniversaries of Dr. Carlos J. Finlay, this author observed what had been published for that purpose and in front of the absence and the silence of several journals (some with the obligation, at least ethics, of remembering to the man), contrasted very pleasantly the praiseworthy editorial that Professor Guillermo Llanos had written years ago in Colombia Médica 3. Without attempting to equal the capacity of synthesis and the style of Professor Llanos; who was recognized with the "Carlos J. Finlay" Order, the highest award offered by the Republic of Cuba to personalities who have contributed exceptionally to scientific development; the author claims, through some elements, to evoke the figure of the eminent scientist on this significant date.

## Development

The phrase of another great man; perhaps the most representative personality of the Cuban public health in that period, with Finlay himself, Juan Guiteras Gener (Matanzas, Cuba 1852-1925), tenacious finlaist and one of the greatest defenders of the sage and his theory; mediator in the dispute arising from the recognition of the discovery between Finlay and members of the US Army Fourth Commission for the Study of the Yellow Fever, is unconcluded to this author: *"... in that occasion there was glory for all... "* 1; yes, certainly he was right, but the really certain thing, was the injustice of robbing of the one that corresponded to someone who neither economic difficulties, or heart disease, neither an oral expression affected as consequence of a difficult to control chorea, neither the typhoid fever, neither the unfriendly negativism that followed per years his discovery, could decrease his scientific battle.

The financial retribution that offered settling down in New York after graduating of medicine at *Jefferson Medical College of Philadelphia* in 1855 2,4, was not enough stimulus to maintain his scientific thought and other imperatives impelled him to exercise in Cuba, for it validates his title in 1857 at the Royal and Literary University of Habana 5, 6.

For his varied scientific activity was seen exercising as great clinical or working on yellow fever, or studying the infantile paralysis, the tetanus of the new born, the tuberculosis, the typhoid fever, the leprosy, the malaria and other diseases that whipped the Cuban population in the XIX century 7. He could be seen reporting illnesses observed for the first time in Cuba 7,8 or writing about tropical illnesses, epidemic disease, bacteriology, or working on Physiologic Pathology, Hygiene and Sanitary Medicine, as well as other medical topics 1,2,6,9 and obviously on Ophthalmology, specialty to which he dedicated several years 10 He also theorized about gravity and other physical and meteorologic problems 11. 

In February of 1881 1,12,13 he proposed in Washington the transmission of the yellow fever for an intermediary agent and in August of that same year, in the Academy of Sciences of the Havana, he read the titled work "The mosquito like agent of transmission of the yellow fever" 14, shattering all the previous theories and formulating a new conception about the infection, based on the role of the vectors in the transmission of illnesses. He was known at a key moment of his existence. The deep emotion that overwhelmed him and the trust in the certainty of his postulates, didn't allow him notice the hostile attitude of the auditory. He thinks that the unbelievers will change to seem when he provides the evidence that support their statements. But he is not able to motivate anybody. When the chairman of the session announces that the word will be granted to those that want to talk about the topic, only the voice of the general secretary of the corporation is listened to request that the work "remains on the table", technical term that indicated that there would not be comments. None of the academics, who attended that day at the auditorium of the Academy, contested the points exposed in the theory of the mosquito *Aedes aegypti* like the transmitting agent of yellow fever, and none was shown of agreement with them. Silence was the only response to a conception that makes it possible the eradication of the so-called "black vomit", and that opened a new chapter in the history of tropical Medicine 1,2.

The years lapsed up to 1900 conformed a period of test for his spiritual and scientific stability. During that period of time, unquestionably long, he maintained a constant and singular battle for the defense and recognition of his theory. Those were truly hard years for his sensitive temperament and his faith and scientific tenacity; years in that his experimental perseverance filled of results, collided rudely with the negative impassibility of everybody; and his theoretical and experimental conclusions tenaciously maintained, were considered more or less than as the result of a scientifically feverish and sick mind 1,15. 

He was always disposed to explain to others the basis of his theory, without trying to hide the difficulties that he had encountered; and unable to convince, not deprived of showing his most natural and exquisite courtesy. With high spirit, located beyond all ephemeral detail and all inferior arrogance was all the time willing to provide, to those new researchers, all his data, including, as is known, to the members of the US Army Fourth Commission for the Study of the Yellow Fever 1,16.

In those years of exclusion, he counted as unshakable columns, with two of the first finlaist according to this author: Adela Shine and Claudio Delgado. Adela, fair wife, excellent partner and devoted collaborator, with her, Finlay founded an exemplary home, of which three children were born. She shared courageously with him all the necessary sacrifices to fill the unevenness of the family budget in difficult times. While the terrible fight lasted to impose the recognition of the theory, she remained lovingly at his side without shows pessimism or fainting, stimulating to him, warmly in his moments of bitterness and in his economic difficulties, logical consequence of the abandonment of customers, forced by his scientific work. According to his son Carlos 1, no one felt more joy and holy pride with him, the glorious day of his triumph, and no one as she wept pain deeper and sincerer in the sad moment of his death.

Claudio Delgado, a Spanish doctor, was his loyal friend, faithful animator and the only assistant in the worst years. Thanks to him, Finlay got to be named by the Governor of the Island, delegate from Cuba and Puerto Rico to the International Sanitary Conference in Washington where in February 18 of 1881, he released his theory. His union to Finlay can be summarized in the letter that he wrote to the scientific when his doctrine was confirmed by the American Commission; then, with all the authority of the present witness's, told him: Has been you, truly the Christ of the redemptive doctrine of the Yellow Fever and you won't lack doctors and Pharisees detractors, neither the persecutions of the envy, neither the taunt neither the gibe of vain and pretentious....; in short, an entire Calvary that you knew to support with philosophical resignation, stiller, with evangelical meekness, having me the honor of being, with you, sometimes the Cyrenian of this passion and always the consequent disciple, as addicted to the doctrine as to the person of the Master" 17. 

The practical applications of his discovery, made it possible that the tropical regions of America, received the benefits of the immigration with whose energy was born the prosperity of peoples and nations. Despite the rejection, the force of his ideas was such that even in life and even in the middle of the difference, the truth began to be recognized by important exponents of the counterparty. Thus, the General Leonard Wood, MD and then Military Governor of the Island of Cuba, in his government report expressed: "...The confirmation of the doctrine of Dr. Finlay is the biggest step that has been given in medical science after the discovery of Jenner's vaccine, and this single fact is enough to justify the war against Spain" 18.

On the other hand, William Gorgas; medical official that directed the sanitation campaign in Cuba and then went to Panama, where he took charge of the sanitary problem that represented the yellow fever and he finished applying the doctrine of Finlay to facilitate the construction of the canal that united for always to the seas around the Americas; in 1910 wrote to the scientist the following note: 

... "If when we went to Cuba we had followed your instructions, we had obtained in 1899 the same results that were achieved later in 1901, and as I believe, thanks to your work and your self-defense of the theory of the mosquito, the American Commission, of which Reed was president, was taken to investigate the theory of the mosquito and without your work, in 1900, the American Commission has never undertaken the investigation of the mosquito theory" 19. 

Finlay was nominated for the Nobel Prize for Physiology and Medicine in 1904 by Dr. Sir Ronald Ross of the Liverpool School of Tropical Medicine; in 1905 by Dr. John W. Ross of the United States Navy, director of "Las Ánimas" (Infectious Diseases Hospital of Havana) during the first US intervention, and in 1907 by Dr. Carl Sundberg, a member of the Prize Committee. For the version of 1912, by Professor Braut Paes Lewe, of the Faculty of Medicine of Rio de Janeiro, and the Academy of Sciences of Havana and for the same year he was proposed with Dr. Agramonte by Dr. Laveran who repeated his proposal for the awards in 1913, 1914 and 1915 1,6,12. Although with exceptional rivals, perhaps the mistake of a grant committee deprives him of the award, although neither with a lot, the most important. Any way, he had risen for always to the height of men like Koch, Pasteur, Golgi, Ramon y Cajal, Laveran, Carrell, Richet and Robert Bárány who had been finally the recognized personalities.

Still after his death, the combat for the amendment continued and the error didn't stop to be repaired; the scientific thought of Finlay was earlier than that of his time, for that reason, in the Pan American Medical Congress of Dallas, Texas, in 1933, for initiative of Dr. Horacio Abascal, it was recognized the day of his birth (3rd of December) each year, as the *Day of the American Medicine* 15,20,21.

In spite of the gibe, and in a majority way, when coming closer to him the men that spoke the same Spanish that English, they discovered that in fact, all spoke the same language, the ideal and immortal language of the science. 

Because your convictions and your universality; critics of those who tried to gnaw the pedestal that only wisdom, perseverance, love and altruism had placed your name; we offer to you, true genius, the clean and unequivocal recognition for your brilliant services to the Science and the Humanity. This it is the biggest prize.
